# Five-Year Outcome of Laparoscopic Fundoplication in Pediatric GERD Patients: a Multicenter, Prospective Cohort Study

**DOI:** 10.1007/s11605-020-04713-4

**Published:** 2020-07-22

**Authors:** Rebecca K. Stellato, Nadia Colmer, Stefaan H. A. Tytgat, David C. van der Zee, Femke A. van de Peppel-Mauritz, Maud Y. A. Lindeboom

**Affiliations:** 1 Department of Biostatistics, Julius Center for Health Sciences and Primary Care, University Medical Center Utrecht, Internal mail no Str. 6.131, P.O. Box 85500, 3508 GA Utrecht, The Netherlands; 2 Department of Pediatric Surgery, Wilhelmina Children’s Hospital, University Medical Center Utrecht, Utrecht, The Netherlands; 3 Department of General Practice, Julius Center for Health Sciences and Primary Care, University Medical Center Utrecht, Utrecht, The Netherlands

**Keywords:** Pediatric, Children, Reflux, GERD, Quality of life, Anti-reflux surgery

## Abstract

**Background:**

Gastroesophageal reflux disease (GERD) is a common disease in children. When drug treatment fails, laparoscopic anti-reflux surgery (LARS) is considered. Short-term follow-up studies report high success rates; however, few studies report long-term results. The aim of this study was to describe the long-term effects of LARS in pediatric patients.

**Methods:**

A prospective, multicenter study of 25 laparoscopic fundoplication patients was performed. At 3 months and 1, 2, and 5 years postoperatively, patients and caregivers were asked to complete the gastroesophageal reflux symptom questionnaire to assess symptoms and the PedsQL™ to assess health-related quality of life (HRQoL).

**Results:**

Reflux symptom severity was still significantly improved 5 years after LARS compared with preoperative levels (*p* < 0.0001). However, 26% of patients reported moderate or severe reflux symptoms. Dysphagia was reported in 13% of patients 5 years after LARS and was more common in children with neurologic impairment and children who underwent a Nissen procedure. The increase in HRQoL 3 months postoperatively appears to decline over time: 5 years after surgery, HRQoL was lower, though not significantly, than 3 months postoperatively. HRQoL at 5 years was still higher, though also not significantly, than preoperative levels. The presence of reflux symptoms after surgery was not significantly associated with lower HRQoL.

**Conclusions:**

LARS is effective for therapy-resistant GERD in children. Five years after surgery, reflux symptoms are still improved. However, we observed a decline in symptom-free patients over time. The initial increase in HRQoL shortly after LARS appears to decline over time.

**Trial Registration:**

Dutch national trial registry Identifier: 2934 (www.trialregister.nl)

**Electronic supplementary material:**

The online version of this article (10.1007/s11605-020-04713-4) contains supplementary material, which is available to authorized users.

## Introduction

Numerous short-term studies on anti-reflux surgery (ARS) in children have been published.^1^–^4^ However, well-designed prospective studies and long-term follow-up data are limited. A wide range in outcome after ARS has been reported, with short-term success rates of 57–100%^1^ and long-term rates of 51–96%.^5^–^12^ This range may be caused by heterogeneous groups, different surgical techniques, and different definitions of success. For example, some studies define the presence of reflux symptoms as a failure, whereas others define only the need for redo surgery as a failure. Moreover, most studies do not use validated questionnaires to adequately assess reflux symptoms at follow-up.^1^,^13^

Since GERD and ARS have a substantial influence on the lives of both patients and caregivers, it is important to evaluate not only reflux symptoms but also on the pre- and postoperative health-related quality of life (HRQoL) of patients.^3^,^14^ Several studies have reported sustained increases in HRQoL in adults,^15^–^17^ but because the etiology of reflux in children is different from that of adults, results from studies in adults cannot be directly generalized to a pediatric population.^3^ Though the majority of pediatric studies has described reflux and reflux associated symptoms, only a few studies have reported on short-term (1 to 6 months) HRQoL of the patients.^2^,^3^,^14^ Studies with longer follow-up have used interviews with, or questionnaires for, adults 5,18,19 or have reported on parent-reported “well-being” 19,20 To our knowledge, no long-term studies of HRQoL after laparoscopic anti-reflux surgery (LARS) have been reported.

The aim of this study is to report the 5-year follow-up of a prospective, multicenter cohort of 25 pediatric patients who underwent LARS. Reflux and dysphagia symptoms and HRQoL are examined longitudinally using validated questionnaires, and potential predictors of LARS failure at 5 years are investigated.

## Materials and Methods

### Study Design

In a prospective, multicenter cohort study, 25 patients were included from 3 hospitals in the Netherlands: Wilhelmina Children’s Hospital, University Medical Centre Utrecht (UMCU); Sophia’s Children’s Hospital, Erasmus University Medical Centre (EMC); and Maastricht University Medical Hospital (MUMC). Patients were included between July 2011 and December 2013 and were operated between August 2011 and May 2014. All pediatric patients aged 2–18, with proton pump inhibitor resistant GERD, were eligible for inclusion. Patients with previous esophageal or gastric surgery (except gastrostomy placement) and patients with structural abnormalities (except esophageal hiatal hernia) were excluded.

### Surgical Procedure

All patients from UMCU underwent an anterior, partial (Thal) fundoplication, whereas patients from EMC and MUMC underwent a posterior, total (Nissen) fundoplication. All fundoplications were performed laparoscopically by experienced pediatric surgeons. Details on the surgical procedure have been published previously.^4^

### Clinical Assessment

Patients had been assessed preoperatively and 3 months after surgery using stationary manometry, 24-h multichannel intraluminal impedance pH monitoring, and a 13C-labeled Na-octanoate breath test. GE half-time percentiles were calculated using reference values from a Dutch population (unpublished chapter of the dissertation of van den Driessche, M, University of Leuven. 2001); GE percentiles higher than 75% were considered delayed. Further details of the clinical assessment have been reported previously.^4^ Patients and caregivers had also been asked to complete two questionnaires preoperatively and 3 months and 1, 2, and 5 years postoperatively.

This article extends previous research on this cohort. The short-term follow-up (3–4 months) and intermediate term follow-up (1–2 years) after ARS have been described in previous papers.^4^,^21^

### Reflux Symptom Questionnaire

The validated, age-adjusted gastroesophageal reflux symptom questionnaire (GSQ) 22 was used to assess reflux and dysphagia symptoms; when patients were older than 18 years of age at 5-year follow-up, the reflux disease questionnaire (RDQ) 23 was used instead. With the GSQ symptoms were scored for frequency in the last 7 days and severity ranging from not at all (1) to most (7) severe. The RDQ uses slightly different symptoms, and scores range from 0 (none) to 5 (daily/most severe). Reflux symptoms were scored as heartburn (two items), regurgitation (two items), and vomiting. Dysphagia was scored as swallowing problems or pain during swallowing. Reflux and dysphagia from both questionnaires were scored as no symptoms, mild (mild symptoms weekly), moderate (mild symptoms daily or severe symptoms weekly), or severe (severe symptoms daily).

### ARS Failure

Failure was defined as the need for redo fundoplication and/or recurring or persistent moderate to severe reflux symptoms according to the GSQ/RDQ.

### Quality of Life Questionnaire

The PedsQL 4.0 Generic Core Scales was used to assess the health-related quality of life (HRQoL).^24^ For patients aged 4–18 years and with normal neurological development (NN), both patients and caregivers completed a questionnaire. For patients under 4 years of age and/or neurologically impaired patients (NI), only caregivers filled in the questionnaire, and for NN patients older than 18 years, only a self-report was completed. The language used in the PedsQL is age adjusted (ages 2–4, 5–7, 8–12, and 13–18). Four domains are scored with the PedsQL, i.e., physical, emotional, social, and school functioning. The scores of the emotional, social, and school functioning are summarized into a psychosocial score. The total score is a summary of all four domains. The scores per domain and the summary scores are converted to a scale from 0 to 100, where a higher score indicate a better HRQoL.

### Ethical Approval and Trial Registration

This study was registered at the start of the study in the Dutch national trial registry (www.trialregister.nl; Identifier: 2934). Ethical approval was obtained from the University Medical Center Utrecht Ethics Committee, and local approval was obtained by the two other participating centers. Informed consent from the patients’ caregivers and patients (≥ 12 years) was obtained prior to study procedures.

### Statistical Analysis

Continuous variables were expressed as mean and standard deviation/error of the mean and categorical variables as number and percentage.

To identify predictors of failure 2 years after LARS, a logistic regression including two pre-specified variables (preoperative GE percentile and age at time of operation) was used. Because two procedures were used in this study, and because previous studies identified NI as a potential predictor,^9^,^20^ two additional analyses were performed: the associations of failure with type of fundoplication and with NI were examined using 95% confidence intervals.

Mixed effects models were used to analyze patterns of symptoms and HRQoL over time. Mixed models account for correlation of repeated measures within a patient and allow for estimation of effects in the presence of missing outcomes over time.^25^ To test the patterns of reflux and dysphagia over time, ordinal logistic mixed models were used. In both models, fixed effects were used for time since operation (categorical), type of fundoplication (Thal or Nissen), and neurological status (NN or NI), and a random intercept per patient was used to account for repeated measures. These models estimate odds ratios (ORs) for increasing symptom severity.

Because the HRQoL scales are continuous, linear mixed models were used to examine the HRQoL over time and relate it to potential explanatory variables. These models estimate differences/changes in HRQoL for the explanatory variables. The outcome for each model was the HRQoL score (total score, physical health subscore, or psychosocial subscore). Both patients’ and caregivers’ reports (when available) were analyzed in one model. Potential explanatory variables (fixed effects) included were gender, age at time of operation, type of fundoplication (Thal or Nissen), neurological status (NN or NI), presence of preoperative delayed gastric emptying, presence of moderate or severe reflux symptoms, time since operation (categorical), and type of report (patient vs. caregiver). A random intercept per patient was used, together with a continuous first-order autoregressive correlation matrix for the residuals to account for repeated measures at unevenly spaced time points.

In order to determine whether patients with NI, preoperative delayed GE, or presence of moderate or severe reflux symptoms have different patterns of HRQoL (total score) over time, the interaction of follow-up time with NI, preoperative delayed GE, or presence of reflux symptoms was added to the mixed model and tested with a likelihood ratio test.

A *p* value < 0.05 was considered statistically significant. All analyses were performed using R version 3.5.1.^26^

## Results

LARS was performed in 25 patients between 2011 and 2013. Baseline characteristics of the participants are displayed in Table 1. The mean age at the time of surgery was 7.3 years (range 2–18). One patient was missing during the 1- and 2-year follow-up but responded again for the 5-year follow-up. Two patients were lost to follow-up at 5 years: a neurologically impaired male who had undergone a Thal fundoplication and a neurologically normal female who had undergone a Nissen fundoplication. At the final follow-up, 23 patients completed the GSQ (*N* = 19) or RDQ (*N* = 4), 19 patients completed the PedsQL, and 17 caregivers completed the PedsQL. The mean follow-up time was 5.2 years (range 4.3–6.4 years).

**Table 1:** Characteristics of the 25 pediatric patients who underwent LARS

Age at time of operation, years–mean ± SD	7.3 ± 4.7
Male sex	13/25 (52.0%)
Neurodevelopmentally impaired	5/25 (20.0%)
Type of fundoplication	
Thal	18/25 (72.0%)
Nissen	7/25 (28.0%)
Preoperative delayed gastric emptying	13/24 (54.2%)
Gastrostomy preoperatively	4/25 (16.0%)
Duration of follow-up, years–mean ± SD	5.2 ± 0.4

### Symptoms

**Fig. 1:**
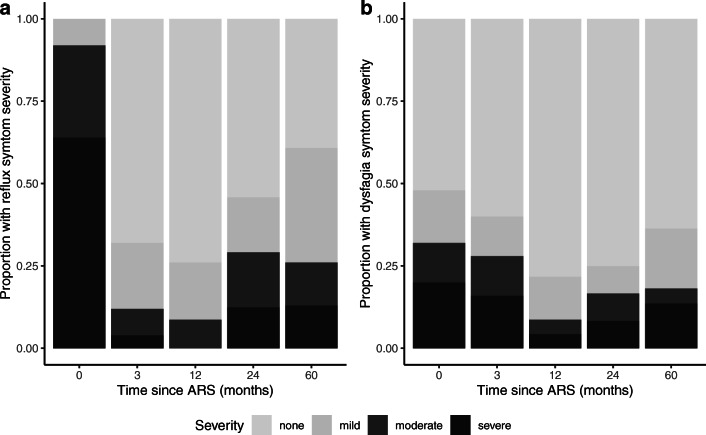
Pre- and postoperative (**a**) reflux and (**b**) dysphagia symptoms for the cohort of 25 pediatric patients who underwent LARS. Symptoms are categorized as none, mild, moderate, and severe

Reflux symptom severity was still significantly improved 5 years after LARS compared with preoperative levels (*p* < 0.0001). Five years after LARS, 26% of the patients reported moderate or severe reflux symptoms compared with 12% at 3 months (Fig. 1a, *p* = 0.0076). The type of fundoplication and neurologic impairment were not statistically significantly associated with reflux severity (*p* = 0.7299 and 0.1431, respectively).

Three of the 23 patients (16%) reported moderate to severe dysphagia symptoms 5 years postoperatively (Fig. 1b). Two patients with moderate dysphagia symptoms after 2 years resolved to no or mild dysphagia symptoms after 5 years, while two other patients without dysphagia symptoms 2 years after LARS reported new-onset dysphagia at 5 years. All three patients with reported dysphagia 5 years postoperatively also reported having reflux symptoms, and all three had undergone a Nissen fundoplication.

Dysphagia symptom severity 5 years after LARS was not significantly improved compared with severity preoperatively or 3 months postoperatively (*p* = 0.3488 and *p* = 0.8144, respectively). In fact, dysphagia severity only was significantly improved 1 and 2 years after LARS compared with preoperative levels (*p* = 0.0156 and *p* = 0.0365, respectively). Children with Nissen fundoplication had an increased chance of more severe dysphagia than children with Thal (OR = 7.1, *p* = 0.0014), as did NI compared with NN children (OR = 21.0, *p* < 0.0001).

### Failure

The failure rate after 2 years, including two redo procedures, was 8/24 (33%). Between 2 and 5 years postoperative, no new redo procedures were necessary. At 5-year follow-up, there was a failure rate of 30%. The decrease in reflux symptoms and failure rate is due to two patients without a redo who reported reflux symptoms (and were therefore considered “failures”) 2 years postoperatively, but reported no or mild reflux symptoms after 5 years. Including these two patients as failures after 5 years results in a cumulative failure rate of 39%. The two patients lost to follow-up at 5 years had no reported reflux symptoms after 2 years.

No variables were found to be significantly predictive of failure. The odds ratio (OR) for age was 1.02 per year (95% CI (0.98–1.07)), and the OR for GE was 1.00 per GE-percentile (95% CI (1.00–1.01)). NI children had a slightly higher percentage of failure than NN (40% vs. 35%, a difference of 5%, 95% CI (− 30.6 to 45.6%)) and children with a Nissen procedure higher than Thal (42.9% vs 33.3%, a difference of 9.6%, 95% CI for (− 25.9 to 45.9%)).

### HRQoL

As reported previously, HRQOL increased significantly in this cohort 3 months after LARS but decreased after 2 years, though not significantly.^14^,^21^ The 5-year PedQL total score is higher, though not significantly, than preoperative levels (mean difference 4.8, 95% CI (− 2.5; 12.0)) and lower (also not significantly) than 3-month postoperative levels (mean difference − 4.3, 95% CI (− 11.6; 2.9)).

**Fig. 2:**
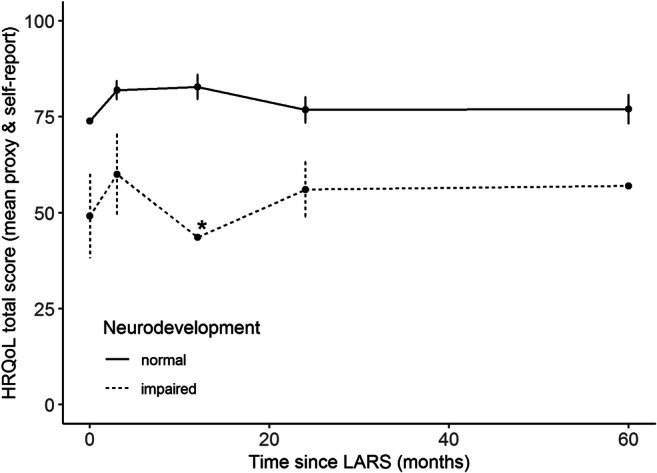
Mean HRQoL (PedsQL total scores) over time for the 20 children with normal neurodevelopment (mean of caregiver proxy report and child self-report) and 5 children with neurological impairment (caregiver proxy report only). Points are means; bars indicate 1 standard error. *It was not possible to calculate a standard error for this mean because only one caregiver returned the questionnaire

Estimated effects of other potential predictors of HRQoL (total score and two subscores) can be found in Supplementary Table 3. NI patients scored on average 25.0 (11.1; 38.8) points lower on the total score compared with NN patients (Fig. 2). Male gender, Thal fundoplication, and a younger age at the time of operation resulted in higher mean HRQoL, though these differences were not statistically significant. Children with delayed GE preoperatively scored 9.7 points lower than those without delayed GE, though again this was not statistically significant, 95% CI (− 20.4; 1.1). Patterns were similar for the physical and psychosocial health subscores (Table 2).

**Table 2:** Mean (standard deviation) of pre- and postoperative HRQoL, as measured by the PedsQL (total scores, physical health, and psychosocial health subscores) for the 25 pediatric patients who underwent LARS

	Preoperatively	3 months PO	1 year PO	2 year PO	5 year PO
Patients	*N* = 12	N = 12	N = 12	*N* = 15	N = 19
Tot	75.8 (8.8)	81.5 (12.8)	81.8 (12.5)	74.4 (16.8)	77.1 (17.8)
PH	81.3 (11.3)	88.3 (13.2)	85.9 (20.3)	77.1 (23.7)	80.8 (21.0)
PS	74.0 (10.4)	79.2 (14.9)	80.5 (11.7)	73.5 (17.1)	75.7 (19.1)
Caregivers	*N* = 25	*N* = 24	*N* = 13	*N* = 20 or 21^*^	*N* = 17
Tot	67.5 (18.5)	77.8 (16.9)	81.0 (17.1)	73.0 (16.8)	71.5 (19.7)
PH	69.5 (25.3)	80.0 (20.7)	80.2 (27.3)	72.0 (29.1)	71.8 (28.3)
PS	66.8 (18.1)	77.0 (17.2)	81.7 (14.3)	74.6 (14.1)	71.8 (18.5)

Patient self-report (neurologically normal children aged 8 and older) and caregiver report (neurologically impaired children and all children under 18 years of age)

*PH* physical health; *PS* psychosocial health; *Tot* total score; *PO* postoperatively

^*^N = 20 (PS) 21(PH, Tot)

For the HRQoL total score, no statistically significant interactions were found between time and NI (*p* = 0.7721), time and preoperative delayed gastric emptying (*p* = 0.6478), and time and reflux symptoms (*p* = 0.2418). This indicates that these characteristics were not significantly associated with a different pattern of HRQoL over time.

## Discussion

The 5-year failure rate of 30% reported in this study falls within the range of 10–43% reported in previous studies.^5^,^6^,^9^,^10^,^27^ The differences in failure rate across studies are likely caused by the lack of a uniform definition of success or failure of ARS. Some studies define failure by the presence of reflux symptoms, with different definitions of severity between studies, while others define failure only by redo fundoplication. The recurrence of reflux symptoms is assessed in some studies using objective measures, such as pH evaluation,^5^,^9^ and sometimes with questionnaires or interviews.^19^ Moreover, the questionnaires used in most studies have not been validated for children. This study is one of few studies that use validated questionnaires for assessment of both symptoms and HRQoL in a pediatric population.

None of the four variables examined was found to be a significant predictor of failure in this cohort. Two previous studies had found potentially elevated risk of GERD recurrence in NI children;^9^,^20^ we saw virtually no difference in the proportions for NI and NN children. However, confidence intervals were wide and compatible with clinically meaningful differences in either direction.

Three months after LARS, only 12% of the patients reported persistent moderate or severe reflux symptoms. After 2 years, that percentage increased to nearly 30% and remained stable 5 years after LARS. An initial good short-term success rate of ARS, with a decline in effect in the long term, is in line with previous studies in children.^7^,^10^,^12^

The presence of moderate or severe dysphagia decreases postoperatively. Five years after LARS, 13% of the patients reported moderate or severe dysphagia, and all patients with reported dysphagia 5 years postoperatively also reported having reflux symptoms. Since reflux esophagitis is associated with dysmotility of the esophagus,^28^ ongoing and late-onset dysphagia is likely to be a manifestation of recurrent reflux.

There was a clinically meaningful increase in HRQoL 3 months and 1 year postoperatively. Two and five years after LARS, the HRQoL is decreased and, while still higher than preoperative levels, does not differ significantly from either pre- or postoperative levels. This is in contrast with previous studies, which reported a significant improvement in the quality of life both 6 months and 4 years after ARS in children 5 or high levels of patient “well-being” several years after surgery.^19^,^20^ However, in those studies, caregivers were asked only one question on the child’s “overall quality of life” or “well-being,” and in two 5,19 selective drop-out may also have biased results.

Three variables were examined for differing patterns of HRQoL over time. While NI patients reported a lower HRQoL at all times, they followed a similar pattern over time compared with NN patients; this is similar to a previous finding.^9^ Children with delayed preoperative GE did not have a different pattern in HRQoL over time than those with normal GE. And although children with and without reflux symptoms appeared to have diverging patterns of HRQoL over time, there was neither a significant interaction between reflux and time nor a significant difference in mean HRQoL averaged over all time points.

The current study has several limitations. As with many studies of pediatric ARS, the sample size is small. To compensate for the resulting limited power, we included one preoperative and four postoperative measurements per child on both symptoms and HRQoL and used statistical methods that make efficient use of all available data and allow for valid estimation in the presence of missing data. Results of mixed effects models are less likely to be affected by selective dropout than analyses on complete cases (such as paired *t* tests or repeated measures ANOVA).^25^ The small sample may nevertheless have limited our ability to detect clinically meaningful differences, as reflected in some confidence intervals.

A second limitation is the use of two different surgical techniques for fundoplication. Consistent with the recommendations of Esposito et al.,^8^ the type of procedure depended on the expertise of the participating center. Consequently the majority of children in this study was operated using a partial (Thal) fundoplication, while less than a third had a complete (Nissen) fundoplication. This is not expected to affect the results; previous studies have examined differences between partial and complete fundoplication and found similar success or reflux control rates between the two procedures.^8^,^29^,^30^ However, we did see more, and more severe, dysphagia among children who underwent a Nissen procedure; this is consistent with a previous finding in children^7^ and with results in adults undergoing LARS.^31^

Finally, following the preoperative and 3-month postoperative clinical assessments, the longer term follow-up in this study included only questionnaires to assess reflux symptoms. The use of questionnaires is less invasive than clinical examination, and the questionnaires were validated and easy to use. However, the questions may be interpreted differently by patients than by clinicians, resulting in over- or underestimation of both reflux and dysphagia. The presence of the assessed symptoms depends on multiple factors, such as comorbidity (such as obstipation) and the use of medication (such as anti-epileptics). A clinical examination could better determine whether symptoms are truly due to GERD or have another root cause.

## Conclusions

Guidelines for the treatment of pediatric GERD recommend conservative use of LARS and clear communication to patients and/or caregivers regarding the potential benefits and risks associated with LARS.^32^ Our results lead us to the same conclusion. After initial short-term improvement of reflux symptoms shortly after LARS, we found an increase in symptoms over time that appeared to stabilize after 2 years of follow-up; the 5-year prevalence of GERD symptoms was still significantly lower than before LARS. Similarly, following a short-term increase, HRQoL declines and then appears to remain stable 2 to 5 years after surgery.

## Electronic supplementary material

ESM 1 (DOCX 14.3 kb).
