# Supplementary material for: Five-Year Outcome of Laparoscopic Fundoplication in Pediatric GERD Patients: a Multicenter, Prospective Cohort Study
Source: J Gastrointest Surg. 2020 Jul 22;25(6):1412–8. doi: 10.1007/s11605-020-04713-4 (PMC8203546; doi:10.1007/s11605-020-04713-4)
Supplement: Supplementary file 1 — (DOCX 14.3 kb). [file 11605_2020_4713_MOESM1_ESM.docx]

# SUPPLEMENTARY TABLES

**Supplementary Table 3**. Estimated parameters from linear mixed model analysis of caregiver proxy and child self-reported PedsQL total, physical health, and psychosocial scores during complete follow-up time.

|  | Total Score | | Physical Health Subscore | | Psychosocial Health Subscore | |
| --- | --- | --- | --- | --- | --- | --- |
| Variable | Estimate [95% CI] | p-value | Estimate [95% CI] | p-value | Estimate [95% CI] | p-value |
| 3 months postoperatively | 9.1 [5.8; 12.5] | <0.0001 | 9.2 [4.2; 14.3] | 0.0004 | 9.0 [5.3; 12.7] | <0.0001 |
| 1 year postoperatively | 9.0 [3.7; 14.3] | 0.0011 | 7.6 [0.1; 15.1] | 0.0481 | 9.4 [3.8; 15.1] | 0.0013 |
| 2 year postoperatively | 3.5 [-1.5; 8.5] | 0.1658 | -0.8 [-7.8; 6.2] | 0.8245 | 5.3 [-0.1; 10.6] | 0.0532 |
| 5 year postoperatively | 4.8 [-0.4; 10.0] | 0.0696 | 1.8 [-5.4; 8.9] | 0.6278 | 5.9 [0.5; 11.3] | 0.0337 |
| Male gender | 6.4 [-4.8; 17.5] | 0.2461 | 3.9 [-9.6; 17.3] | 0.5551 | 7.6 [-4.1; 19.3] | 0.1865 |
| Neurologically impaired | -25.7 [-38.8; -11.1] | 0.0013 | -44.7 [-61.6; -27.9] | <0.0001 | -17.7 [-32.2; -3.2] | 0.0196 |
| Nissen fundoplication | -3.0 [-16.7; 10.8] | 0.6560 | -9.1 [-25.6; 7.4] | 0.2607 | -0.6 [-14.9; 13.8] | 0.9318 |
| Age at operation (years) | -0.3 [-1.7; 1.1] | 0.6638 | -0.6 [-2.3; 1.1] | 0.4729 | -0.2 [-1.7; 1.3] | 0.7762 |
| Child report (vs. Caregiver) | -0.5 [-4.6; 3.6] | 0.8214 | 0.5 [-5.0; 6.0] | 0.8594 | -0.8 [-5.0; 3.3] | 0.6902 |
| Preoperative delayed GE | -9.3 [-20.4; 1.1] | 0.0746 | -11.3 [-24.2; 1.7] | 0.0836 | -9.2 [-20.5; 2.0] | 0.1024 |

CI = confidence interval; GE = gastric emptying
